# A Hydrogel Delivery System Based on Selenium Nanoparticles and bFGF for Promoting the Repair of Skin Wounds

**DOI:** 10.3390/biomedicines14061401

**Published:** 2026-06-22

**Authors:** Yue Wang, Ruoyang Chen, Chaoqun Wang, Pei Zheng, Min Chen, Huihui Lu

**Affiliations:** 1 Department of Nursing, Affiliated Renji Hospital, School of Medicine, Shanghai Jiao Tong University, Shanghai 200120, China; 2 Department of Urology, Affiliated Renji Hospital, School of Medicine, Shanghai Jiao Tong University, Shanghai 200120, China

**Keywords:** hydrogel, selenium, skin wounds, inflammation

## Abstract

**Objectives:** Skin wound repair has long remained a crucial clinical challenge, in response to which, in this study, we propose a novel injectable hydrogel delivery system. In particular, we focus on the efficient delivery of bioactive factors and modulation of the local wound microenvironment. **Methods:** The hydrogel integrates selenium nanoparticles (SeNPs) and basic fibroblast growth factor (bFGF), which serve as key therapeutic components in the proposed system, and are additionally co-integrated with oxidized hyaluronic acid (OHA) and heparin-grafted carboxymethyl chitosan (CMCS-g-Hep) to construct a multifunctional SeNPs/bFGF-loaded CMCS-g-Hep/OHA hydrogel network. Accordingly, this proposed hydrogel was systematically evaluated using chemical synthesis, physicochemical characterization, in vitro cellular assays, and C57BL6J mice studies, which we used to jointly assess the biocompatibility and wound-healing efficacy of the proposed system. **Results:** The results demonstrated that the hydrogel enabled sustained bFGF release and was capable of significantly enhancing fibroblast proliferation, migration, and collagen deposition. In a mouse skin defect model, treatment with the loaded hydrogel markedly accelerated wound closure. Additionally, we conducted mechanistic investigations to further illustrate that the hydrogel can modulate the wound microenvironment by regulating inflammatory and chemotactic signaling pathways. **Conclusions:** These findings suggest a promising therapeutic pathway for chronic wound repair.

## 1. Introduction

Globally, skin wounds have emerged as a major challenge for healthcare systems [1], a phenomenon that is particularly pronounced among patients undergoing major surgical procedures [2,3]. These persistent wounds not only prolong hospitalization and elevate the likelihood of reoperation but may also precipitate secondary infections and graft dysfunction or loss, thereby severely compromising patients’ physical recovery and psychological well-being [4]. Moreover, wound sites are particularly susceptible to complex microbial colonization, leading to a chronic inflammatory microenvironment that disrupts normal fibroblast and keratinocyte functions and further impairs tissue regeneration [5,6]. Consequently, difficult-to-heal wounds represent a challenging clinical bottleneck in postoperative recovery management and long-term patient prognosis.

Currently, clinical approaches to promote wound healing include the following strategies: routine dressing changes, negative-pressure wound therapy, topical biological agents, and tissue-engineering-based materials [7,8]. Among these strategies, the synergistic integration of bioactive factors, such as basic fibroblast growth factor (bFGF), with advanced biomaterials has become a heated research field [9,10,11,12]. BFGF is essential in chronic wound repair due to its outstanding performance in stimulating fibroblast proliferation and enhancing collagen synthesis [13]; however, its clinical efficacy is substantially limited by rapid in vivo degradation and a short biological half-life, limitations which render sustained therapeutic activity difficult to achieve when bFGF is administered alone. In addition, oxidative stress and persistent inflammatory responses within the chronic wound microenvironment are also key obstacles to effective healing. In recent years, nanomaterials such as selenium nanoparticles (SeNPs) have attracted increasing attention owing to their favorable biocompatibility and pronounced antioxidant and anti-inflammatory activities [14,15,16]. Despite these advantages, most existing delivery platforms remain functionally simplistic, with limited capacity to simultaneously achieve sustained release, microenvironmental modulation, and practical clinical applicability. As a result, their therapeutic performance remains suboptimal for skin wounds. Thus, this field requires novel delivery systems capable of coordinated, multifunctional bioactive regulation to be developed, which constitutes an urgent technical challenge.

Against this backdrop, in the present study we propose a novel strategy to construct a CMCS-g-Hep/OHA/SeNPs/GF hydrogel delivery system, in which SeNPs and bFGF function as a synergistic bioactive core. Meanwhile, injectable, sustained-release oxidized hyaluronic acid (OHA) and heparin-grafted carboxymethyl chitosan (CMCS-g-Hep) serve as the encapsulating matrix, which carries the previous two substances. SeNPs possess favorable antioxidant, anti-inflammatory, and biocompatible properties, allowing effective suppression of oxidative stress and chronic inflammatory responses within the wound microenvironment. In parallel, the hydrogel exhibits suitable mechanical integrity and tunable degradability, while facilitating the sustained release of bFGF to preserve its long-term bioactivity. Collectively, this novel strategy is expected to enhance the overall therapeutic efficacy of chronic non-healing wound repair.

## 2. Materials and Methods

### 2.1. Synthesis and Characterization of SeNPs

SeNPs were synthesized via vitamin C (Vc)-mediated reduction of sodium selenite. Briefly, 10 mL of a 20 mM Na_2_SeO_3_ solution was mixed with bovine serum albumin (BSA), followed by the dropwise addition of a 60 mM Vc solution under continuous stirring. The reaction mixture gradually changed from colorless to orange-red, indicating SeNP formation. The yielded colloidal solution was maintained under stirring at room temperature in the dark for 3 h to allow complete nucleation and growth, and unreacted ions were subsequently removed using dialysis. The resulting products were collected by centrifugation and redispersed to obtain a homogeneous stock suspension. The hydrodynamic particle size distribution and polydispersity index (PDI) were determined using dynamic light scattering (DLS), after which the zeta potential was used to evaluate surface charge characteristics. The morphology of SeNPs was examined using transmission electron microscopy (TEM, Hitachi, Singapore); surface functional groups were analyzed using Fourier transform infrared spectroscopy (FT-IR, Thermo Scientific, Waltham, MA, USA); and the crystalline structure was characterized using X-ray diffraction (XRD).

### 2.2. Preparation of Hydrogels

OHA was prepared as follows: Briefly, 1.0 g of hyaluronic acid (HA) was dissolved in deionized water, and sodium periodate (NaIO_4_) was then added under light-shielded conditions for selective oxidation. The reaction was allowed to proceed at room temperature for 12 h to introduce aldehyde functionalities. Upon completion, ethylene glycol was added to quench the oxidation reaction and the resulting solution was purified by dialysis against deionized water to remove residual reagents, yielding OHA with a tunable degree of oxidation suitable for subsequent crosslinking.

CMCS-g-Hep was synthesized via the following procedure: To impart growth factor-binding capability to the material, heparin was covalently grafted onto the side chains of carboxymethyl chitosan (CMCS) via carbodiimide-mediated coupling using 1-ethyl-3-(3-dimethylaminopropyl)carbodiimide (EDC) and N-hydroxysuccinimide (NHS). Briefly, CMCS was dissolved in MES buffer and subsequently combined with a pre-activated heparin solution. The reaction mixture was stirred at room temperature for 24 h to form amide bonds between the carboxyl groups of heparins and the amino groups of CMCS. The resulting CMCS-g-Hep conjugate was dialyzed and then lyophilized for further use.

To quantitatively determine the substitution degree of heparin, we employed the Toluidine Blue colorimetric assay. By measuring the unreacted heparin in the dialysate, we calculated the grafting efficiency. The results demonstrate that distinct heparin grafting signals are detectable in CMCS-g-Hep, confirming successful incorporation of heparin into the CMCS molecular chain following the EDC/NHS coupling reaction.

A growth factor (bFGF) stock solution (20 μg/mL) was gradually titrated into the CMCS-g-Hep solution to achieve a final concentration of 400 ng/mL. This process enables a [CMCS-g-Hep–bFGF] affinity complex to form through specific heparin–bFGF interactions. Subsequently, an SeNP suspension was introduced into the complex solution to obtain a dual-functional precursor containing both freely dispersed SeNPs and affinity-bound bFGF. An OHA solution was then mixed with the precursor at a 1:1 (*v*/*v*) ratio to serve as the crosslinking component. Gelation occurred in situ through dynamic Schiff base reactions between OHA aldehyde groups and CMCS-g-Hep amino groups, resulting in the loaded gel formation. The OHA exhibits an oxidation degree of approximately 40%, indicating that sufficient aldehyde groups have been successfully introduced into the HA molecular chain, enabling subsequent formation of a dynamic Schiff base crosslinking network with CMCS-g-Hep. The obtained hydrogels were freeze-dried and stored at −20 °C.

Through this approach, optimal gelation formulations were preliminarily determined using macroscopic observation. The internal microstructure and porous morphology of the freeze-dried hydrogels were examined using scanning electron microscopy (SEM); injectability was evaluated using extrusion testing through a 26 G needle; rheological properties, including temperature-dependent behavior and shear-thinning characteristics, were assessed using temperature sweep and shear-thinning measurements; and mechanical properties, such as compressive strength and modulus, were determined using compression testing.

We adopted a sterile preparation approach rather than terminal sterilization. Specifically, all precursor solutions (OHA, CMCS-g-Hep, and bFGF) were separately dissolved and sterilized via filtration through 0.22 μm sterile polyethersulfone (PES) syringe filters prior to gelation. The SeNP suspension was synthesized using sterile water and similarly filtered/handled under sterile conditions. All subsequent mixing and hydrogel fabrication steps were meticulously performed under strictly aseptic conditions within a Class II biological safety cabinet.

### 2.3. Cell Experiments

The human dermal fibroblasts (HSF cells) used in this study were purchased from BeNa Culture Collection (Product No.: BNCC341540), and were derived from the HFF-1 cell line of ATCC, with the accession number SCRC-1041. The HFF-1 cell line (ATCC SCRC-1041) is a fibroblast cell isolated from the foreskin of a donor, which can be used to produce feeder cells. All the purchased HSF cells were authenticated by STR profiling to ensure their correctness and purity before being used in the experiment. HSFs were cultured in Dulbecco’s modified Eagle’s medium (DMEM) supplemented with 10% fetal bovine serum (FBS), 100 U/mL penicillin, and 100 μg/mL streptomycin at 37 °C in a humidified atmosphere containing 5% CO_2_. Cells in the logarithmic growth phase were collected for all subsequent experiments.

#### 2.3.1. In Vitro bFGF Release Study

An appropriate amount of loaded gel was placed into a dialysis bag and immersed in 5 mL of phosphate-buffered saline (PBS, pH 7.4). The system was incubated at 37 °C under gentle shaking (100 rpm) and at predetermined time points (0–72 h), 1 mL of the release medium was collected and replaced with an equal volume of fresh PBS. The concentration of released bFGF was quantified using an enzyme-linked immunosorbent assay (ELISA), and cumulative release profiles were subsequently plotted.

#### 2.3.2. Transwell Migration Assay

HSFs were divided into four groups, namely control, bFGF, loaded gel co-culture, and blank hydrogel co-culture. Cells were pretreated for 48 h prior to the migration assay and diluted Matrigel was applied to the upper chambers of Transwell inserts and allowed to polymerize. Subsequently, serum-free HSF suspensions were seeded into the upper chambers, while the lower chambers were filled with DMEM containing 10% fetal bovine serum (FBS) as a chemotactic stimulus. After 24 h of incubation, the migrated cells on the membrane lower surface were fixed and stained with crystal violet. Cells in three randomly selected fields were counted under a microscope.

Scratch (Wound-Healing) Assay: HSFs were seeded into 6-well plates and cultured until confluence was reached. After a scratch was created, the cells were then cultured with media corresponding to each treatment group or with hydrogel extracts. Images of the scratch area were captured at 0, 24, and 48 h using an optical microscope and the percentage of wound closure was quantified using ImageJ software (1.54f).

#### 2.3.3. Western Blot Analysis

After 48 h of treatment, HSFs from each group were harvested, and total cellular proteins were extracted using RIPA lysis buffer. Protein concentrations were determined using a bicinchoninic acid (BCA) assay. Equal amounts of protein were separated by sodium dodecyl sulfate–polyacrylamide gel electrophoresis (SDS-PAGE) and subsequently transferred onto polyvinylidene fluoride (PVDF) membranes, which were blocked with 5% (*w*/*v*) skim milk and incubated overnight at 4 °C with primary antibodies against collagen type I alpha 1 (Col1a1), transforming growth factor-β1 (TGF-β1), α-smooth muscle actin (α-SMA), and β-actin as the internal control. After incubation with horseradish peroxidase (HRP)-conjugated secondary antibodies, protein bands were visualized using enhanced chemiluminescence (ECL), with band intensities quantified using ImageJ software.

### 2.4. Animal Experiments

#### 2.4.1. Animals and Wound Model Establishment

Specific pathogen-free (SPF) male C57BL/6 mice (6–8 weeks old, 20–25 g) were selected for the animal experiments in this study. Following anesthesia, the dorsal hair of the mice was shaved, their skin was disinfected, and a difficult-to-heal skin model was established using an 8 mm biopsy needle. All animal experiments were approved by the Ethics Committee of Renji Hospital, School of Medicine, Shanghai Jiao Tong University.

The mice were randomly assigned to four groups: (1) model group (saline), in which wounds were treated daily with saline; (2) bFGF group, in which wounds received daily local injections of bFGF solution; (3) loaded gel group, in which wounds were covered with loaded gel daily; (4) blank gel, in which wounds were treated with blank hydrogel daily. Treatments were administered throughout the experimental period and digital images of the wounds were captured on postoperative days 0, 3, 6, 9, 12, 15, 18, and 21. Wound areas were quantified using ImageJ software, with closure rates calculated accordingly.

#### 2.4.2. Tissue Collection and Histological Analysis

Mice were euthanized on postoperative day 21, and wound tissues, along with adjacent normal skin, were harvested. Tissue samples were fixed in 4% paraformaldehyde, embedded in paraffin, and sectioned at a thickness of 5 μm. Hematoxylin and eosin (H&E) and Masson’s trichrome staining were performed to evaluate granulation tissue formation and collagen deposition under a light microscope.

Cytokine Measurement: Supernatants from homogenized wound tissues were collected and analyzed using ELISA kits to determine the concentrations of the pro-inflammatory cytokines interleukin-6 (IL-6) and tumor necrosis factor-α (TNF-α), as well as the anti-inflammatory cytokine interleukin-10 (IL-10).

### 2.5. RNA-Seq and Bioinformatics Analysis

RNA sequencing (RNA-seq) was conducted on mouse skin tissues, with each experimental group containing three independent biological replicates. The purity and quantity of each mRNA sample were assessed by the Nanodrop 2000 spectrophotometer manufactured by Thermo Scientific, Waltham, MA, USA. Libraries for RNA were created utilizing the Illumina^®^ Strandedm RNAPrep, Ligation kit. MAJORBIO Biotech Co., Ltd. (Shanghai, China) performed transcriptome sequencing and analysis, generating paired-end reads using the Illumina NovaSeq X Plus platform(V2.1.0). RNA-seq data quality was assessed using RSeQC (v2.6.4). To identify DEGs, DESeq2 was employed. Significant DEGs were determined using a threshold of adjusted *p* value < 0.05 along with a fold change >2 or <0.5. GO enrichment analysis for DEGs was performed through the hypergeometric distribution using David bioinformatics to pinpoint genes that were significantly enriched.

### 2.6. Statistical Analysis

All data are analyzed using SPSS version 22.0 software. Quantitative results are presented as mean ± standard deviation (mean ± SD) and comparisons among multiple groups are performed using one-way analysis of variance (ANOVA). For pre-planned comparisons (e.g., each experimental group vs. control), Dunnett’s test is used; for all other pairwise comparisons, Tukey HSD is applied to ensure a conservative approach. *p* < 0.05 is considered statistically significant.

## 3. Results

### 3.1. Synthesis and Characterization of SeNPs

**Figure 1:**
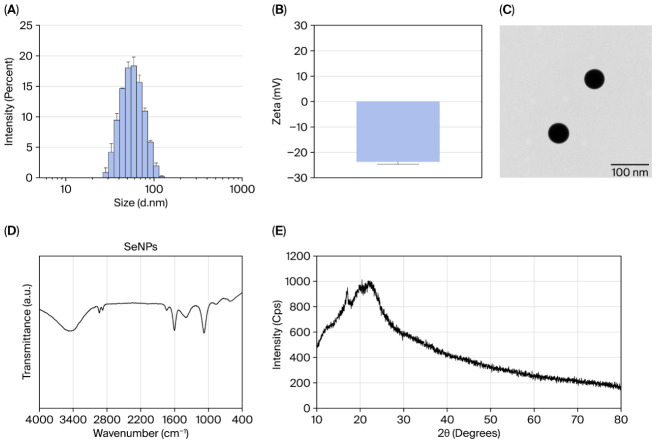
Physicochemical characterization of the synthesized selenium nanoparticles (SeNPs). (**A**) SeNPs have a mean diameter of 55.51 ± 2.24 nm and a PDI of 0.16 ± 0.01. (**B**) Zeta potential measurement indicates a surface charge of −26.97 ± 0.64 mV. (**C**) Representative TEM micrograph of the SeNPs. (**D**) FT-IR spectrum displaying bands corresponding to O–H stretching (~3400 cm^−1^), H–O–H bending (~1600 cm^−1^), and Se–O/Se–Se vibrations (<1000 cm^−1^). (**E**) XRD pattern confirming a trigonal crystalline phase, with peak broadening indicative of nanoscale crystallinity. Scale bar: 100 nm. Data are presented as mean ± SD (n = 3).

Nanoparticle size potentiometer analysis demonstrated that the synthesized SeNPs exhibited a unimodal and narrow hydrodynamic size distribution, with a mean particle size of 55.51 ± 2.24 nm and a polydispersity index (PDI) of 0.16 ± 0.01 (Figure 1A), indicating good dispersion and minimal aggregation. Zeta potential measurements showed that the SeNPs carried a negative surface charge (−26.97 ± 0.64 mV), which is conducive to colloidal stability by providing sufficient electrostatic repulsion between particles (Figure 1B). SEM further confirmed the nanoparticles’ morphological features (Figure 1C), while Fourier transform infrared (FT-IR) spectroscopy revealed a broad absorption band around 3400 cm^−1^, corresponding to O–H stretching vibrations. A weak band near 1600 cm^−1^ was attributed to the adsorbed water bending vibration (H–O–H), while the weak absorption below 1000 cm^−1^ was tentatively assigned to Se–O or Se–Se vibrational modes (Figure 1D). These spectral features suggest that SeNPs possess a hydrophilic surface with a thin surface oxide layer. XRD analysis showed that the SeNPs exhibited a typical trigonal crystalline selenium phase, with distinct diffraction peaks and no detectable impurity-related signals, indicating high crystalline purity (Figure 1E). Notably, the diffraction peaks simultaneously displayed evident broadening, which is characteristic of nanoscale crystalline materials. In summary, these comprehensive characterization results confirm our successful synthesis of SeNPs with uniform morphology, favorable colloidal stability, and a high-purity trigonal crystalline structure.

### 3.2. Physicochemical Properties of Hydrogels

**Figure 2:**
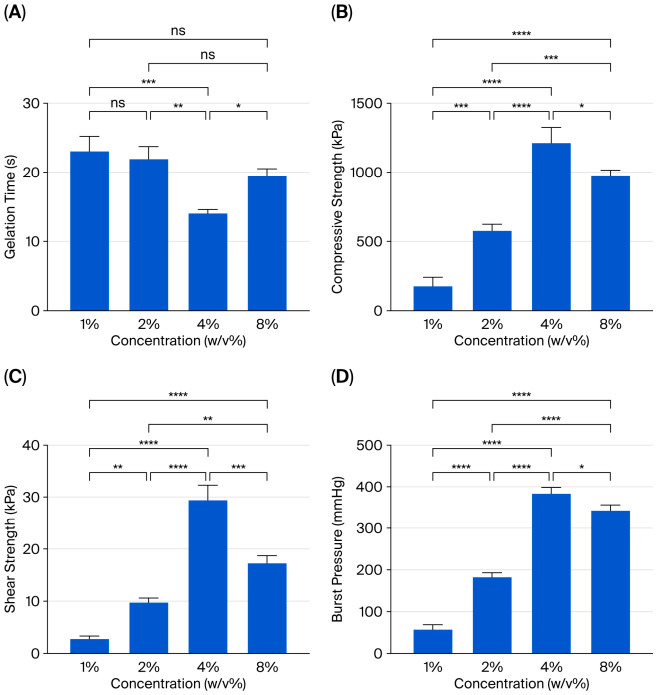
Physicochemical and mechanical characterization of hydrogels at different polymer concentrations. (**A**) Gelation time measurements showing the fastest gelation at a 4% concentration. (**B**) Compressive strength increases with concentration up to 4% then slightly decreases at 8%. (**C**) Shear strength reaches a maximum at 4%, indicating optimal network density and crosslinking. (**D**) Burst pressure comparison with physiological blood pressure. All hydrogel formulations exceed physiological levels, with 4% and 8% showing notably higher pressure resistance. Data are presented as mean ± SD (n = 3). Statistical significance is indicated as ns: not significant; * *p* < 0.05; ** *p* < 0.01; *** *p* < 0.001; and **** *p* < 0.0001.

In order to optimize gelation behavior, loaded gel precursor solutions were prepared at different polymer concentrations (1%, 2%, 4%, and 8%, *w*/*v*). At the specific concentrations of 1% and 2%, the hydrogels exhibited relatively long gelation times, while increasing the concentration to 4% resulted in a favorable balance between injectability and rapid gel formation, with a gelation time of approximately 10–15 s (Figure 2A). In contrast, a further increase to 8% led to a prolonged gelation process, likely due to restricted molecular mobility at higher polymer densities. The compressive strength increased from approximately 200 kPa at 1% concentration to around 600 kPa at 2%, reaching a maximum of approximately 1200 kPa at 4%. At 8%, the compressive strength slightly decreased to about 1000 kPa (Figure 2B). Hydrogels at 1% concentration exhibited a shear strength of approximately 5 kPa, which increased to about 10 kPa at 2% and peaked at approximately 30 kPa at 4%. At 8%, the shear strength decreased to around 20 kPa (Figure 2C). This trend indicates that the internal network of the 4% hydrogel achieves an optimal structural density, where enhanced polymer chain entanglement and an increased number of effective crosslinking points collectively improve shear deformation resistance. This section also illustrates the comparison between burst and physiological pressure conditions; the former increased progressively with hydrogel concentration from 1% to 8%. Importantly, the burst pressures of all tested formulations exceeded normal physiological blood pressure (≈120 mmHg), indicating sufficient mechanical robustness for wound closure or tissue sealing applications (Figure 2D). Notably, the 4% and 8% hydrogels exhibited substantially higher burst pressures than physiological levels, suggesting strong resistance to pressure fluctuations in dynamic biological environments. Based on a comprehensive evaluation of gelation kinetics, injectability, and mechanical performance, we selected the 4% hydrogel formulation for subsequent experiments.

### 3.3. Physicochemical Properties of SeNP- and bFGF-Loaded Hydrogels

**Figure 3:**
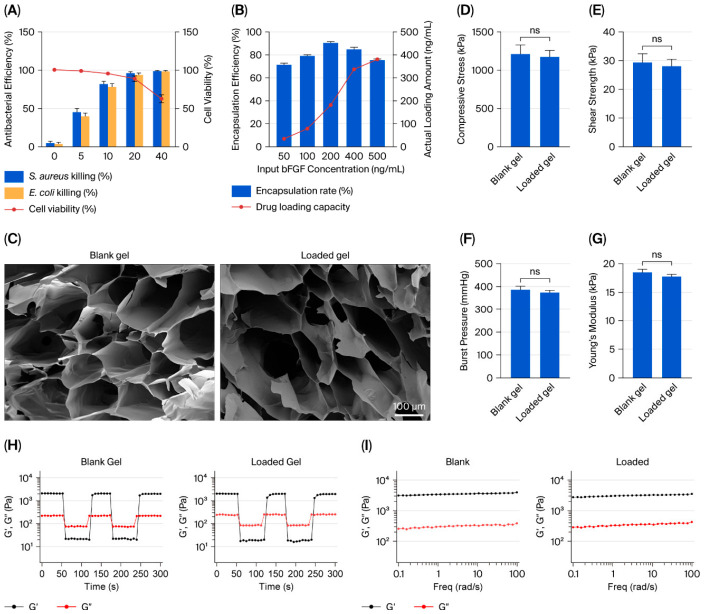
Optimization and characterization of SeNP- and bFGF-loaded hydrogels. (**A**) Determining the optimal SeNP concentration based on the balance between antibacterial efficacy and cytocompatibility. A concentration of 20 μg/mL was selected. (**B**) Selection of the optimal bFGF loading concentration based on encapsulation efficiency and drug-loading capacity. (**C**) SEM image of the lyophilized loaded hydrogel cross-section, showing a uniform, interconnected, honeycomb-like porous architecture. (**D**–**F**) Comparison of mechanical properties between blank and loaded hydrogels: (**D**) compressive strength, (**E**) shear strength, and (**F**) burst pressure. Both formulations show comparable performance, with burst pressures exceeding physiological blood pressure. (**G**) Representative compressive stress–strain curves showing that the loaded hydrogel maintains excellent ductility. (**H**,**I**) Rheological properties, (**H**) storage (G′) and loss (G″) moduli over time, and (**I**) the mechanical spectrum (G′ and G″ versus angular frequency), demonstrating stable elastic solid behavior (G′ > G″). Scale bar: 100 μm. Data are presented as mean ± SD (n = 3). Statistical significance is indicated as ns: not significant.

SeNP concentration markedly influenced both the antibacterial activity and cytocompatibility of the hydrogel system; as the SeNP concentration increased from 0 to 40 μg/mL, the antibacterial efficacy against Staphylococcus aureus and Escherichia coli increased correspondingly, reaching a bactericidal rate exceeding 95% at concentrations ≥20 μg/mL. In parallel, cell viability gradually declined with increasing SeNP content, with a pronounced reduction observed at 40 μg/mL. Considering the balance between antimicrobial effectiveness and acceptable cytocompatibility, an SeNP concentration of 20 μg/mL was selected for subsequent experiments (Figure 3A). Meanwhile, a bFGF concentration of 400 ng/mL provided optimal encapsulation efficiency and drug-loading capacity and was therefore chosen as the loading dose (Figure 3B). Afterwards, we assessed the core physicochemical properties of the SeNP- and bFGF-loaded hydrogel (loaded gel), and compared them with those of the blank hydrogel (blank gel). Scanning electron microscopy of lyophilized hydrogel cross-sections revealed a uniform, interconnected, honeycomb-like porous architecture with pore sizes ranging from 100 to 150 μm (Figure 3C). Compression, shear, and burst pressure tests demonstrated comparable mechanical performance between the two formulations, with burst pressures in both cases far exceeding normal physiological blood pressure, indicating that incorporating bioactive components did not harm mechanical integrity or pressure resistance (Figure 3D–F). Although Young’s modulus of the loaded gel was slightly reduced, stress–strain analysis revealed only marginally lower stress values and excellent ductility, with no fracture observed during compression. Rheological analysis showed that the loaded gel storage modulus (G′) remained consistently higher than the loss modulus (G″), indicating stable elastic solid behavior (Figure 3H,I). These findings indicate that functional components enhanced the effective crosslinking density and structural stability of the hydrogel network. During a 14-day in vitro degradation study, the loaded gel exhibited a significantly slower degradation rate and higher mass retention compared with the blank gel (Supplementary Figure S1A). Both SeNPs and bFGF were released in a sustained and continuous manner over the same period without an initial burst release; still, their release profiles differed (Supplementary Figure S1B). In addition, the DPPH radical scavenging assay showed that the loaded gel possessed significantly higher antioxidant activity than the blank gel (Supplementary Figure S1C). Meanwhile, the hydrogel was found to have good injection properties (Supplementary Figure S1D). Overall, the optimized loaded gel exhibits a favorable porous structure, good injectability, robust mechanical and rheological stability, sustained-release behavior, and integrated antimicrobial and antioxidant functionalities.

### 3.4. BFGF-Loaded Hydrogel Promotes HSF Migration and Functional Protein Expression

**Figure 4:**
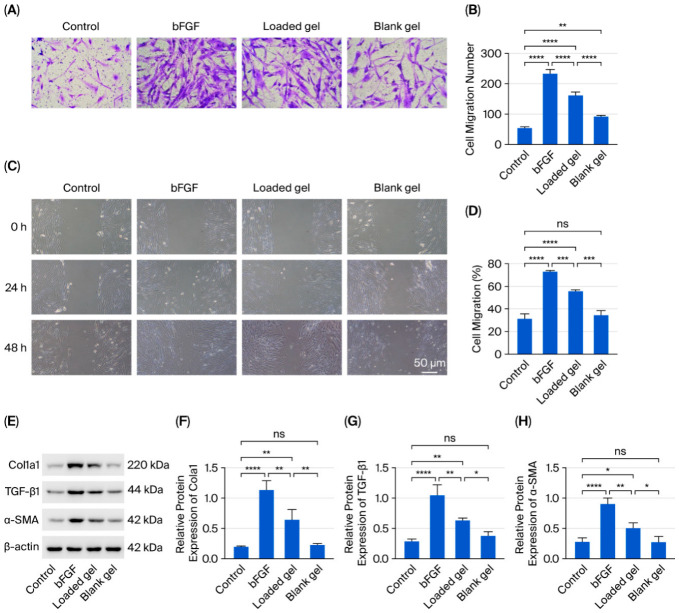
bFGF-loaded hydrogel promotes HSF migration and upregulates functional protein expression. (**A**) Representative images of HSF migration assessed by Transwell assay. (**B**) Representative images of HSF migration assessed by scratch wound healing assay. (**C**,**D**) Quantitative analysis of cell migration from Transwell and scratch assays, respectively. (**E**) Representative Western blot bands showing Col1a1, TGF-β1, and α-SMA expression levels. (**F**–**H**) Quantitative analysis of Col1a1, TGF-β1, and α-SMA protein expression levels. Scale bar: 50 or 100 μm. Data are presented as mean ± SD (n = 3). Statistical significance is indicated as ns: not significant; * *p* < 0.05; ** *p* < 0.01; *** *p* < 0.001; and **** *p* < 0.0001.

To assess the effects of the loaded gel on the migratory behavior of HSFs, we performed Transwell migration and scratch assays. The bFGF and loaded gel groups exhibited significantly enhanced HSF migration compared with the control and blank gel groups (Figure 4A–D). Further analysis of protein expression levels for Col1a1, TGF-β1, and α-SMA revealed that both the bFGF and loaded gel groups exhibited significantly elevated expression of Col1a1, TGF-β1, and α-SMA compared with the control group (Figure 4E–H). The bFGF group demonstrated higher expression levels than the loaded gel group, while the TGF-β1 and α-SMA expression levels in the bFGF group increased remarkably compared with those in the control group. The expression levels in the bFGF group were higher than those in the loaded gel group, consistent with the migration assay results, further validating the biological activity of bFGF. Collectively, these results indicate that bFGF enhances HSF migration and the expression of key functional proteins involved in extracellular matrix synthesis and wound repair. While the hydrogel carrier alone did not induce significant cellular responses, the bFGF-loaded hydrogel enabled controlled delivery of the bFGF and effectively modulated fibroblast migration and collagen-related signaling pathways, supporting its potential application in wound healing and tissue regeneration.

### 3.5. BFGF-Loaded Hydrogel Promotes Wound Repair in Mice

**Figure 5:**
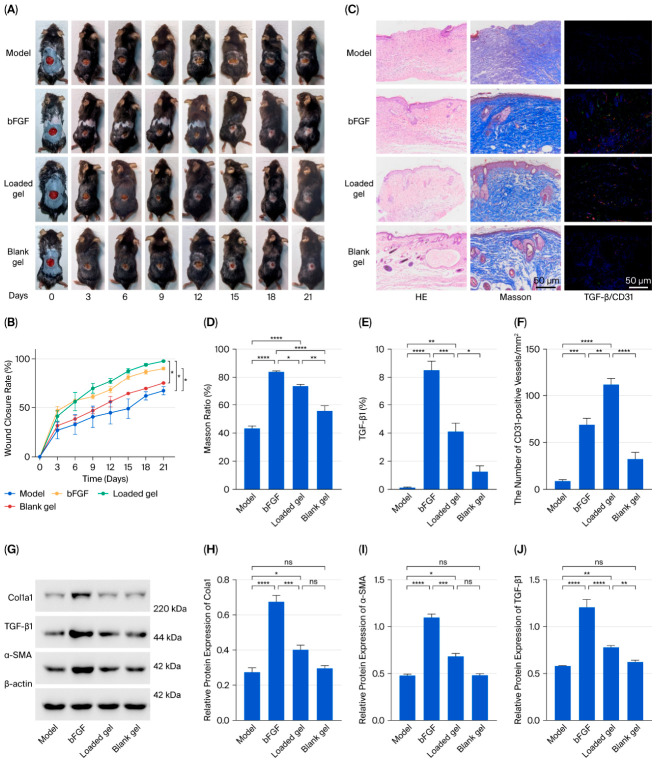
bFGF-loaded hydrogel promotes wound repair in a skin defect model. (**A**) Schematic timeline of the in vivo study. (**B**) Representative macroscopic images of wound closure over 18 days in the four treatment groups: model (saline), bFGF injection, blank gel, and loaded gel. (**C**–**F**) Representative histological and immunofluorescence images and quantitative analysis of wound tissues harvested on day 21: (**C**) H&E and Masson’s trichrome staining and TGF-β1/CD31 immunofluorescence staining for microvessels. (**D**–**F**) Quantitative analysis. (**G**) Representative Western blot bands showing the Col1a1, TGF-β1, and α-SMA expression levels in wound tissues. (**H**–**J**) Quantitative analysis of Col1a1, TGF-β1, and α-SMA protein expression levels. Scale bar: 50 μm. Data are presented as mean ± SD (n = 6). Statistical significance is indicated as ns: not significant; * *p* < 0.05; ** *p* < 0.01; *** *p* < 0.001; and **** *p* < 0.0001.

In this study, we established a skin defect model on the dorsal region of C57 mice for further investigation. Animals were randomly assigned to four groups: saline-treated control (model group), local bFGF injection group, loaded gel group, and blank gel. Macroscopic wound healing was monitored over time, with the model group exhibiting the slowest wound closure throughout the observation period. Although the bFGF injection group showed accelerated wound closure during the early stage, the healing rate declined at later time points. In contrast, the loaded gel group demonstrated a consistently higher healing rate beginning on day 6. By day 18, wounds in this group were nearly completely epithelialized and exhibited more uniform contraction, suggesting enhanced re-epithelialization and tissue remodeling. Histological and immunofluorescence analyses of wound tissues collected on day 21 further revealed differences in repair quality among the groups (Figure 5A,B): the model group displayed loosely arranged granulation tissue, disorganized collagen fibers, low-CD31^+^ microvascular density, and weak TGF-β1 expression, while the bFGF group showed increased collagen deposition and partial neovascularization. Nevertheless, elevated TGF-β1 expression suggested a tendency toward excessive fibrotic responses. In comparison, the loaded gel group exhibited a dense and well-organized collagen architecture, significantly increased CD31^+^ vascular density, and markedly reduced TGF-β1 expression relative to the bFGF group, indicating enhanced angiogenesis accompanied by restrained fibrosis (Figure 5C–F). These histological findings were further supported by WB analysis. Compared with the model group, both the bFGF injection and loaded gel groups showed increased Col1a1, TGF-β1, and α-SMA expression (Figure 5G–J). Surprisingly, the expression levels of all three proteins were significantly lower in the loaded gel group than in the bFGF group, suggesting that sustained bFGF release via hydrogel promotes wound repair-related signaling while mitigating excessive fibrotic activation. Subsequently, the major organs (including the liver, kidney, and spleen) as well as renal function were examined in mice treated with the bFGF-loaded hydrogel. No obvious damage was observed in these organs or in kidney function (Supplementary Figure S2A,B).

### 3.6. Mechanistic Investigation of bFGF-Loaded Hydrogel-Mediated Skin Repair in Mice

**Figure 6:**
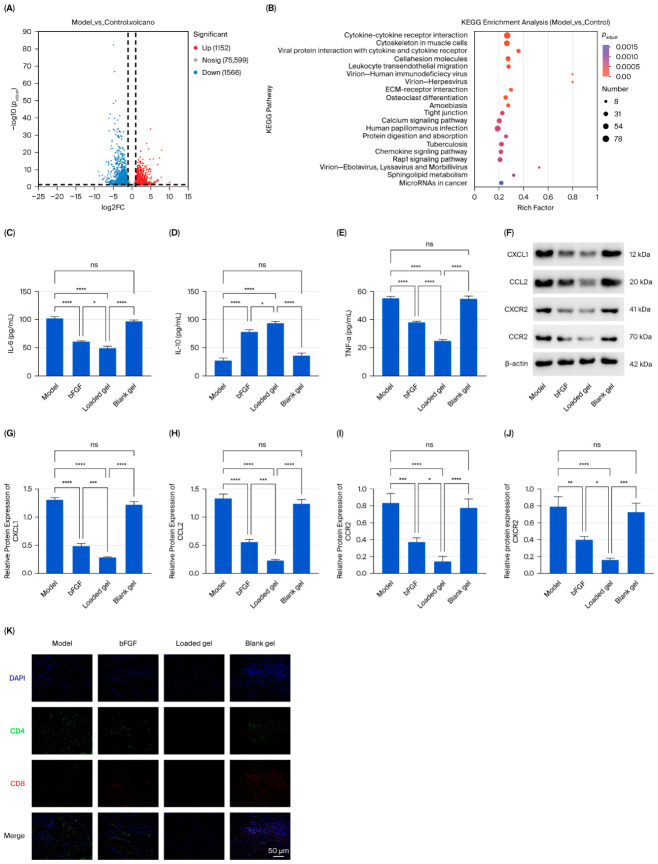
Mechanistic investigation of bFGF-loaded hydrogel-mediated skin repair in mice. (**A**) Volcano plot of differentially expressed genes identified via bioinformatics analysis of wound tissues. (**B**) KEGG pathway enrichment analysis showing significant enrichment of regulated genes in inflammation- and immune-related pathways, such as the chemokine signaling pathway. (**C**–**E**) ELISA quantification of key cytokines in wound tissues: (**C**) IL-6, (**D**) TNF-α, and (**E**) IL-10. (**F**) Representative Western blot bands for chemokine signaling pathway proteins. (**G**–**J**) Quantitative protein expression level analysis for (**G**) CXCL1, (**H**) CCL2, (**I**) CCR2, and (**J**) CXCR2. (**K**) Representative immunofluorescence images showing infiltration of CD4^+^ and CD8^+^ T lymphocytes at the wound site. Scale bar: 50 μm. Data are presented as mean ± SD (n = 6). Statistical significance is indicated as ns: not significant; * *p* < 0.05; ** *p* < 0.01; *** *p* < 0.001; and **** *p* < 0.0001.

We first performed bioinformatics analyses to identify the key regulatory networks involved in wound healing. Volcano plots of differentially expressed genes and KEGG pathway enrichment analysis revealed that a subset of significantly regulated genes were enriched in inflammation- and immune-related pathways such as the chemokine signaling pathway (Figure 6A,B). These results indicate that inflammatory regulation constitutes a central mechanism in skin repair. In order to validate the in vivo immunomodulatory effects of the loaded gel, we quantified cytokine levels in wound tissues. ELISA results showed that loaded gel treatment significantly reduced the expression of pro-inflammatory cytokines IL-6 and TNF-α, while simultaneously increasing the anti-inflammatory cytokine IL-10 (Figure 6C–E). In contrast, the free-bFGF group exhibited a weaker regulatory effect on these inflammatory mediators. These findings suggest that loaded gel more effectively modulates the inflammatory microenvironment toward a repair-favorable state. We further studied the involvement of chemokine-associated signaling pathways at the protein level. WB analysis demonstrated that loaded gel treatment markedly downregulated the wound tissue expression of chemokines CXCL1 and CCL2, as well as their corresponding receptors CXCR2 and CCR2, compared with the control group. This suppression level was significantly more pronounced than that observed in the free-bFGF group (Figure 6F–J). These results indicate that the hydrogel system effectively hinders chemokine signaling axis overactivation. Given the critical role of chemokines in immune cell recruitment, we next assessed immune cell infiltration at the wound site. Immunofluorescence staining revealed substantial infiltration of CD4^+^ and CD8^+^ T lymphocytes in control wounds (Figure 6K), while in contrast, loaded gel treatment significantly reduced the local accumulation of both T cell populations, with a greater inhibitory effect than free-bFGF treatment.

## 4. Discussion

After surgery, patients often experience delayed wound healing due to hypoalbuminemia and concurrent comorbidities, conditions which usually result in persistent inflammation and disruption of the local immune microenvironment [4,17] and under which physiological skin wound healing is markedly compromised. Clinically, this is presented as delayed wound closure, increased susceptibility to infection, and aberrant fibrotic responses. Although current clinical strategies, including pharmacological agents and biomaterial-based dressings, are widely used to facilitate wound repair, the therapeutic efficacy for recipients is lacking. In particular, most approaches fail to simultaneously address immune dysregulation and tissue regeneration, which represents a central challenge in this patient population. Consequently, developing advanced therapeutic platforms capable of precisely modulating local immune responses while providing sustained regenerative support is of substantial clinical significance for improving healing outcomes, reducing postoperative complications, and enhancing quality of life.

In this study, we developed an injectable hydrogel delivery system integrating SeNPs and bFGF, designed to synergistically combine anti-inflammatory regulation with pro-regenerative activity. A series of assessment methods, including comprehensive physicochemical characterization, in vitro functional assays, and in vivo evaluation, collectively demonstrated that this system effectively improves the wound microenvironment. Specifically, the hydrogel enabled stable and sustained release of bFGF, and also attenuated excessive inflammatory cell infiltration through modulating chemokine-related signaling pathways. Compared with free-bFGF injection, the bFGF-loaded SeNP hydrogel system demonstrated superior wound-healing outcomes, which may be attributed to the combined effects of its sustained release, SeNP-mediated redox modulation, and matrix support.

Previous studies have established that surface charge and hydrophilicity are key determinants regulating nanomaterial biological behavior, including their dispersion stability, interactions with cell membranes, and persistence within complex physiological microenvironments [18,19]. The negatively charged surface of SeNPs reduces nonspecific adsorption of plasma proteins and suppresses immune recognition. Moreover, it simultaneously enhances colloidal stability in biological fluids, thereby favoring prolonged in vivo residence [20]. With respect to carrier performance, the loaded gel system combines a highly interconnected porous architecture with excellent injectability, while offering tunable mechanical strength and degradation behavior. Consistent with previous reports, hydrogels featuring pore sizes of 150 μm are particularly conducive to cell migration, nutrient diffusion, and extracellular matrix deposition, thereby facilitating effective tissue regeneration [21,22,23]. Incorporating dynamic covalent crosslinks, such as Schiff base bonds, further enhances the self-healing capacity of the hydrogel and provides a molecular basis for controlled and sustained-release of both GFs and nanoparticles. Additionally, SeNP incorporation not only reinforces the compressive mechanical properties of the hydrogel but also imparts additional biological functions, including antioxidant and anti-inflammatory activity. This coordinated structural integrity and biological functionality enhancement remains uncommon in conventional hydrogel-based systems. Compared with commonly reported bFGF delivery platforms described in previous studies, such as microspheres or membrane-based systems, the present hydrogel demonstrates clear advantages in preserving bFGF bioactivity and achieving smooth, sustained release profiles. Its modular design also allows flexible adjustment of release kinetics to accommodate different therapeutic requirements. Consequently, these features position the loaded gel as a multifunctional and tunable delivery platform realizing methodological innovation in relevant research areas.

Regarding the regulatory effects of bFGF delivery on fibroblast behavior, hydrogel-based systems enable sustained bFGF release while effectively promoting proliferation, migration, and extracellular matrix protein expression of HSF. Previous studies have demonstrated that bFGF exerts dose-dependent effects on fibroblast motility and collagen synthesis, and that the physicochemical properties of delivery carriers critically influence the preservation of its biological activity and duration of action. Free bFGF is prone to rapid inactivation both in vitro and in vivo as a result of enzymatic degradation and nonspecific protein adsorption, leading to transient and unstable biological responses. In contrast, hydrogel-based delivery provides a protective microenvironment that shields bFGF from premature degradation and enables its controlled, sustained release. Prolonged bFGF availability continuously activates downstream signaling pathways, thereby enhancing fibroblast migration dynamics and upregulating functional proteins such as Col1a1, TGF-β1, and α-SMA. It is worth noting that the hydrogel’s interconnected porous architecture and dynamic covalent crosslinked network facilitate cell infiltration and migration while closely recapitulating the biomechanical characteristics of the native extracellular matrix. This biomimetic microenvironment supports more physiologically relevant fibroblast behavior in three-dimensional culture conditions, consistent with previous reports demonstrating the regulatory role of hydrogel microenvironments on cellular fate and function. Comparative analyses among delivery platforms indicate that hydrogels achieve more stable maintenance of bFGF bioactivity and smoother sustained-release profiles than conventional carriers such as microparticles or membrane-based systems, resulting in prolonged cellular responses. In vivo, the loaded gel exhibited significantly enhanced repair efficacy in skin defect models compared with both the control and free-bFGF treatment groups. Mechanistically, this improvement is attributed to coordinated re-epithelialization, angiogenesis, and collagen remodeling regulation. Sustained bFGF release from the hydrogel promotes endothelial cell proliferation and migration while simultaneously modulating the TGF-β1 signaling axis, thereby limiting excessive fibrotic responses and improving the structural and functional restoration of wound tissue. Collectively, these findings demonstrate that the hydrogel delivery strategy extends beyond conventional carrier functions by enabling multidimensional optimization of wound-healing outcomes.

At the mechanistic level, the loaded gel system markedly suppresses the expression of pro-inflammatory cytokines, chemokines, and their corresponding receptors, while concomitantly upregulating the anti-inflammatory cytokine IL-10. Meanwhile, it significantly reduces local immune cell infiltration, including CD4^+^ and CD8^+^ T lymphocytes. These outcomes jointly indicate comprehensive remodeling of the wound inflammatory microenvironment. Accumulating evidence suggests that sustained activation of chemokines such as CXCL1 and CCL2, together with their receptors, plays a central role in perpetuating chronic inflammation and aberrant immune cell recruitment during impaired wound healing [24]. Compared with conventional anti-inflammatory drug delivery strategies, hydrogel-based systems provide a sustained and localized regulatory platform that attenuates excessive pro-inflammatory signaling while promoting immune homeostasis through the gradual release of bioactive factors and microenvironmental modulation, contributing to shortening the inflammatory phase of wound repair and facilitating the transition toward tissue regeneration and remodeling.

## Data Availability

The original data presented in this study are included in the article/Supplementary Material. Data and materials not included in the article are available from the corresponding author upon request.

## Supplementary Materials

The following supporting information can be downloaded at: https://www.mdpi.com/article/10.3390/biomedicines14061401/s1, Figure S1: Additional functional properties of the hydrogels. Figure S2. Biosafety evaluation of the bFGF-loaded hydrogel in mice.

## References

[1] Iversen A.K.S., Lichtenberg M., Fritz B.G., Díaz-Pinés Cort I., Al-Zoubaidi D.F., Gottlieb H., Kirketerp-Møller K., Bjarnsholt T., Jakobsen T.H. (2024). The chronic wound characterisation study and biobank: A study protocol for a prospective observational cohort investigation of bacterial community composition, inflammatory responses and wound-healing trajectories in non-healing wounds. BMJ Open.

[2] Siskind E., Huntoon K., Shah K., Villa M., Blood A.J., Lumerman L., Fishbane L., Goncharuk E., Oropallo A., Bhaskaran M. (2012). Partial closure of skin wounds after kidney transplantation decreases the incidence of postoperative wound infections. Int. J. Angiol..

[3] Nashan B.R., Citterio F. (2012). Wound healing complications and the use of mammalian target of rapamycin inhibitors in kidney transplantation: A critical review of the literature. Transplantation.

[4] Lu H., Zheng P., Chen R., Chen M. (2023). Analysis of risk factors for impaired wound healing after kidney transplantation. Int. Wound J..

[5] Peña O.A., Martin P. (2024). Cellular and molecular mechanisms of skin wound healing. Nat. Rev. Mol. Cell Biol..

[6] Caldwell M.D. (2020). Bacteria and Antibiotics in Wound Healing. Surg. Clin. N. Am..

[7] Ito D., Ito H., Ideta T., Kanbe A., Ninomiya S., Shimizu M. (2021). Systemic and topical administration of spermidine accelerates skin wound healing. Cell Commun. Signal. CCS.

[8] Beraja G.E., Gruzmark F., Pastar I., Lev-Tov H. (2025). What’s New in Wound Healing: Treatment Advances and Microbial Insights. Am. J. Clin. Dermatol..

[9] Zhang J., Li H., Zhang L., Wang J.L. (2019). Observation of curative effect of recombinant human basic fibroblast growth factor combined with compound polymyxin B ointment and local application of insulin on wound healing of deep second-degree burn in diabetes mellitus: A randomized study. Eur. Rev. Med. Pharmacol. Sci..

[10] Geng Y., Gao Y., Qi D., Wang Z., Zou Z., Zhang Z., Lian J., Zhang Z., He C., Shao Y. (2025). A hydrogel tissue adhesive incorporating basic fibroblast growth factor-loaded liposomes accelerates cutaneous wound healing by enhancing cell proliferation, collagen synthesis and angiogenesis. Acta Biomater..

[11] Kumar S., Chu A., Theis T., Rastogi S., Costea D.M., Banerjee R., Das B.C., Yarmush M.L., Hsia H., Cohen R. (2024). Self-Assembled Fibroblast Growth Factor Nanoparticles as a Therapeutic for Oxidant-Induced Neuronal and Skin Cell Injury. ACS Appl. Bio Mater..

[12] Matsumine H., Niimi Y. (2022). Basic fibroblast growth factor-impregnated collagen gelatin sponge completes formation of dermis-like tissue within 2 weeks: A prospective cohort study. Regen. Ther..

[13] Ono I., Akasaka Y., Kikuchi R., Sakemoto A., Kamiya T., Yamashita T., Jimbow K. (2007). Basic fibroblast growth factor reduces scar formation in acute incisional wounds. Wound Repair Regen..

[14] Zhao L., Wang J., Pan Y., Tan F., Wang T., Ran H., Pang M., Zou X., Xu P., Chen A. (2025). Selenium-Albumin Nanoaccelerator Hydrogel Promotes Wound Healing by Antibacterial, Anti-Inflammatory and Antioxidant along with Inhibits Scar Formation via Downregulating CD36. Adv. Healthc. Mater..

[15] Khabibullaeva N., Khaitbaev A., Mansurov D., Khakimov Z., Rakhmanov A., Vidović E., Benassi E. (2025). Bioactive composite based on chitosan obtained from the exoskeleton of dead Apis mellifera and selenium nanoparticles for accelerated skin wound healing: Synthesis, characterisation and in vivo assessment. Int. J. Biol. Macromol..

[16] Chen G., Yang F., Fan S., Jin H., Liao K., Li X., Liu G.B., Liang J., Zhang J., Xu J.F. (2022). Immunomodulatory roles of selenium nanoparticles: Novel arts for potential immunotherapy strategy development. Front. Immunol..

[17] Peluso G., Incollingo P., Campanile S., Menkulazi M., Scotti A., Tammaro V., Calogero A., Dodaro C., Carlomagno N., Santangelo M.L. (2020). Relation Between Wound Complication and Lymphocele After Kidney Transplantation: A Monocentric Study. Transplant. Proc..

[18] Li B., Mao J., Wu J., Mao K., Jia Y., Chen F., Liu J. (2024). Nano-Bio Interactions: Biofilm-Targeted Antibacterial Nanomaterials. Small.

[19] Rodriguez-Lejarraga P., Martin-Iglesias S., Moneo-Corcuera A., Colom A., Redondo-Morata L., Giannotti M.I., Petrenko V., Monleón-Guinot I., Mata M., Silvan U. (2024). The surface charge of electroactive materials governs cell behaviour through its effect on protein deposition. Acta Biomater..

[20] Filipovi N., Ušjak D., Milenkovi M.T., Zheng K., Liverani L., Boccaccini A.R., Stevanović M.M. (2021). Comparative Study of the Antimicrobial Activity of Selenium Nanoparticles with Different Surface Chemistry and Structure. Front. Bioeng. Biotechnol..

[21] Ma Y., Wang X., Su T., Lu F., Chang Q., Gao J. (2022). Recent Advances in Macroporous Hydrogels for Cell Behavior and Tissue Engineering. Gels.

[22] Han Y., Lian M., Wu Q., Qiao Z., Sun B., Dai K. (2021). Effect of Pore Size on Cell Behavior Using Melt Electrowritten Scaffolds. Front. Bioeng. Biotechnol..

[23] Lu D., Cai K., Zeng Z., Huang J., Ma N., Gao B., Yu S. (2025). VEGF loading heparinized hyaluronic acid macroporous hydrogels for enhanced 3D endothelial cell migration and vascularization. Biomater. Adv..

[24] Ahmad T.B., Liu L., Kotiw M., Benkendorff K. (2018). Review of anti-inflammatory, immune-modulatory and wound healing properties of molluscs. J. Ethnopharmacol..

