# Supplementary material for: A Hydrogel Delivery System Based on Selenium Nanoparticles and bFGF for Promoting the Repair of Skin Wounds
Source: Biomedicines. 2026 Jun 22;14(6):1401. doi: 10.3390/biomedicines14061401 (PMC13297135; doi:10.3390/biomedicines14061401)
Supplement: Supplementary file 1 [file biomedicines-14-01401-s001.zip › biomedicines-4336007-supplementary.pdf]

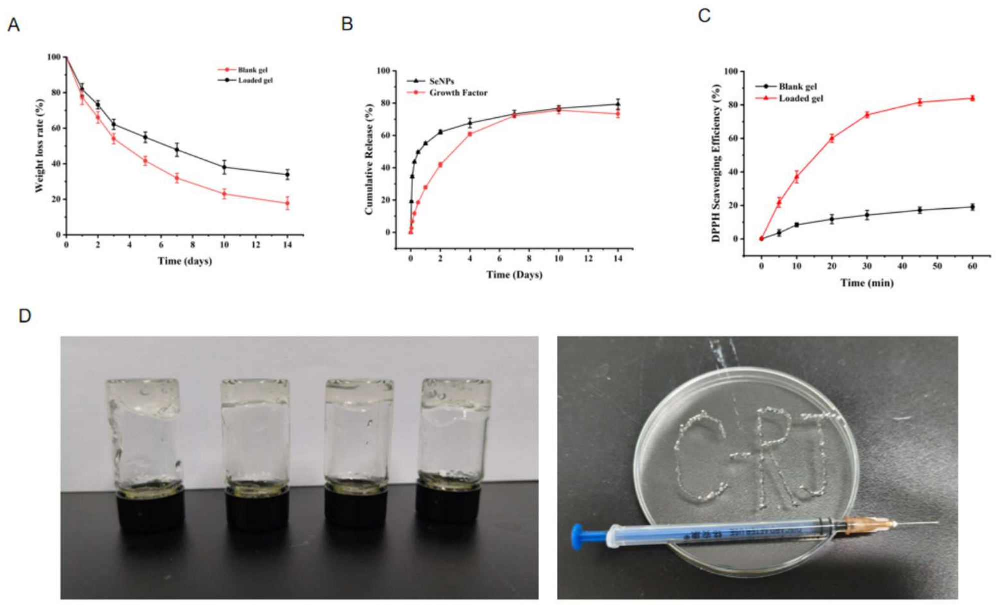

Supplementary Figure S1. Additional functional properties of the hydrogels.

(A) In vitro degradation profiles over 14 days, showing significantly slower degradation and higher mass retention for the loaded hydrogel compared to the blank control.

(B) Cumulative release profiles of SeNPs and bFGF from the loaded hydrogel over 14 days, showing sustained release without an initial burst.

(C) DPPH radical scavenging assay, indicating significantly higher antioxidant activity for the loaded hydrogel.

(D) Demonstration of the good injectability of the hydrogel formulation.

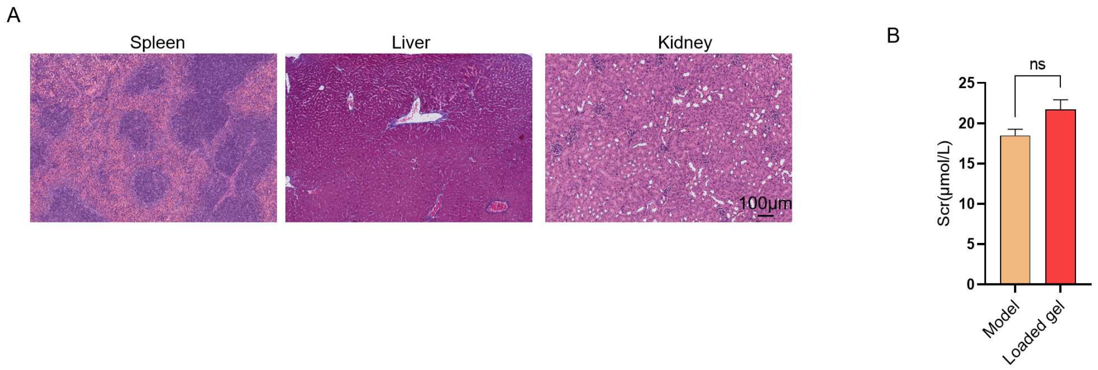

Supplementary Figure S2.Biosafety evaluation of the bFGF-loaded hydrogel in mice.

(A) Histological examination of major organs (liver, kidney, spleen) showing no apparent damage.

(B) Assessment of renal function revealing no significant impairment.

Scale bar: 100 μm.Data are presented as Mean ± SD(n=6). Statistical significance is indicated as ns:not significant ,*p < 0.05, **p < 0.01, ***p < 0.001, ****p < 0.0001.
